# Adverse events of hepatic function disorder in Japanese patients with radically unresectable or metastatic renal cell carcinoma treated with pembrolizumab plus axitinib: a post-marketing surveillance study

**DOI:** 10.1007/s10147-025-02708-2

**Published:** 2025-04-29

**Authors:** Mototsugu Oya, Shotaro Yasuoka, Takuto Tokudome, Toshihiko Minegishi, Masahiro Hamada, Masahiko Ozaki, Shinichiroh Maekawa, Yuichiro Ito

**Affiliations:** 1 Department of Urology, Keio University School of Medicine, Tokyo, Japan; 2 Oncology Medical Affairs, MSD K.K., 1‑13‑12 Kudan‑kita, Chiyoda‑ku, Tokyo, 102‑8667 Japan; 3 Japan Pharmacovigilance, MSD K.K., Tokyo, Japan

**Keywords:** Advanced renal cell carcinoma, Hepatic function disorder, Japan, Pembrolizumab plus axitinib, Post-marketing surveillance

## Abstract

**Background:**

Post-marketing surveillance focusing on hepatic function disorder was requested owing to its higher incidence in the pembrolizumab plus axitinib group than in the sunitinib group in KEYNOTE-426. We aimed to evaluate the prevalence and risk factors of adverse events (AEs) of hepatic function disorder in patients with unresectable/metastatic renal cell carcinoma (RCC) treated with pembrolizumab plus axitinib in real-world clinical practice in Japan.

**Methods:**

Patients were observed for 9 months after starting treatment with pembrolizumab plus axitinib.

**Results:**

In total, 193 patients were included in the safety analysis set (median age, 70 years). Most patients did not have a history of hepatic function disorder before starting treatment (96.4%, 186/193). The median treatment period was 27.1 weeks. At the 9-month data cut-off, 62.2% (120/193) of patients discontinued treatment, the most common reason being any AE in 31.1% (60/193). The incidence of AEs of hepatic function disorder was 30.1% (58/193) for any grade and 15.0% (29/193) for grade ≥ 3. Most AEs of hepatic function disorder occurred within 3 months from starting treatment. AEs of hepatic function disorder were the reason for discontinuation of pembrolizumab in 9.3% (18/193) of patients; axitinib, 7.3% (14/193); and both pembrolizumab and axitinib, 5.2% (10/193). No background factors were identified as being associated with the occurrence of AEs of hepatic function disorder.

**Conclusion:**

There were no new safety signals for AEs of hepatic function disorder, and the incidence was consistent with that reported in KEYNOTE-426, in Japanese patients with radically unresectable/metastatic RCC treated with pembrolizumab plus axitinib.

**Supplementary Information:**

The online version contains supplementary material available at 10.1007/s10147-025-02708-2.

## Introduction

According to Global Cancer Statistics, 431,288 new cases of kidney cancer were diagnosed in 2020, and the number of related deaths was 179,368, accounting for approximately 2% of all new cancer cases and cancer deaths worldwide [1]. According to the Japanese Cancer Registry, 30,458 cases of kidney cancer were diagnosed in Japan in 2019, and the number of related deaths was 9712 in 2020 [2]. In Japan, the incidence and death rates per 100,000 population are 24.1 and 7.9 cases, respectively, both of which are twice as high in men than in women [2]. In general, renal cell carcinoma (RCC) accounts for approximately 90% of cases of kidney malignancy [3], and approximately 20–30% of RCC cases metastasize [4].

Systemic treatments approved for advanced RCC include treatment with a tyrosine kinase inhibitor (TKI) [5–9], immune checkpoint inhibitor (ICI)/TKI combination therapy [10–12], and ICI/ICI combination therapy [13, 14]. Pembrolizumab is a humanized monoclonal antibody of the immunoglobulin G4 subclass against human programmed cell death protein 1 (PD-1). By inhibiting the binding between PD-1 and its ligands, PD-L1 and PD-L2, the activation of cancer antigen-specific T cells and cytotoxic activity against cancer cells is enhanced and tumor growth is suppressed. The combination of pembrolizumab (an ICI) and axitinib (a TKI) has been shown to be an effective and safe treatment for advanced RCC in a randomized, open-label, global phase 3 trial (KEYNOTE-426) [15, 16], with these findings confirmed in a subset of Japanese patients enrolled in KEYNOTE-426 [17]. Based on the results of the KEYNOTE-426 trial, in 2019, pembrolizumab plus axitinib was approved for first-line treatment of patients with advanced RCC in the US, Europe, and Japan [18–20].

Treatment-related adverse events (TRAEs) of hepatic function disorder were identified as adverse events (AEs) of special interest (AEOSI) by the Japan Pharmaceuticals and Medical Devices Agency. In the KEYNOTE-427 trial, which evaluated pembrolizumab monotherapy for clear cell RCC, the incidence of grade ≥ 3 hepatitis, an immune-related AE, was 1.8% [21]. In a post-marketing surveillance (PMS) study of pembrolizumab monotherapy for urothelial carcinoma, the incidence of grade ≥ 3 hepatic function disorder, an AEOSI, was 2.5% [22]. Approximately 25% of patients from large clinical trials on axitinib presented with elevations in serum aminotransferase levels; however, values greater than five times the upper limit of normal (Common Terminology Criteria for Adverse Events [CTCAE] grade 3) were much rarer (1–2% of patients) [23]. In patients enrolled in KEYNOTE-426, the incidence of hepatic function disorder was higher in patients treated with pembrolizumab plus axitinib (all grades: 40.6%, grade ≥ 3: 21.2%) than in patients treated with sunitinib (all grades: 26.6%, grade ≥ 3: 6.1%) [24].

Therefore, this PMS study was requested to evaluate the prevalence and risk factors of AEs of hepatic function disorder in patients with unresectable or metastatic RCC who were treated with pembrolizumab plus axitinib in real-world clinical practice in Japan.

## Patients and methods

### Study design and patients

This was a PMS study conducted at 138 centers in Japan. The survey period was from 30 March 2020 to 26 April 2023 (data cut-off), and the enrollment period was from 30 March 2020 to 31 January 2022. The observation period was 9 months after the initiation of treatment with pembrolizumab plus axitinib. The survey was conducted using Electronic Data Capture with a prospective central registry. The study was conducted in accordance with the Declaration of Helsinki and adhered to the Good Post-marketing Study Practice Ordinance in Japan. The ordinance exempts the requirement of ethical review in PMS studies. The evaluation of the effectiveness of pembrolizumab plus axitinib was out of scope for this PMS study.

All patients with radically unresectable or metastatic RCC who were included in the study were treated with pembrolizumab (200 mg administered by intravenous infusion over 30 min every 3 weeks or 400 mg administered by intravenous infusion over 30 min every 6 weeks) plus axitinib during the enrollment period. The axitinib dose was started at the dose determined by the attending physician and adjusted accordingly.

### Data collection and evaluations

The data collected included the following: the incidence of AEs of hepatic function disorder and TRAEs of hepatic function disorder, the causal relationship to the study drug (causality was determined by the study investigator for each drug as “can be ruled out” or “cannot be ruled out”), the severity of events (graded according to CTCAE version 5-Japan Clinical Oncology Group), serious AEs, the time to onset of events, treatment interventions for AEs of hepatic function disorder, and outcomes of AEs of hepatic function disorder. The following events were considered serious: events that resulted in death, life-threatening events, events that required hospitalization for treatment or prolongation of existing hospitalization, events that resulted in persistent or significant disability or incapacity, events that resulted in birth defects, and other medically significant events or reactions. Of note, if an event was deemed non-serious by the attending physician but serious by the sponsor company’s decision, the event was included as a serious AE for the purpose of this PMS. Risk factors for AEs of hepatic function disorder were also evaluated.

AEs were recorded using the Medical Dictionary for Regulatory Activities/Japanese version preferred term. If the lowest level terms of the AEs were assigned to the same preferred term, these AEs were regarded as the same AE, and if the same AE occurred more than once in the same patient, these events were counted only once.

### Statistical methods

The target sample size was set at 150 patients based on the following: in KEYNOTE-426, the incidence of AEs of hepatic function disorder was 49.4% (212 of 429 patients); and the incidence of grade ≥ 3 AEs of hepatic function disorder was 21.2% (91 of 429 patients) [25]. Therefore, a sample size of 150 patients was considered sufficient to observe approximately 30 patients with grade ≥ 3 AEs of hepatic function disorder.

The analysis population of the present study was the safety analysis set, defined as all patients who received at least one dose of pembrolizumab plus axitinib and for whom the presence or non-presence of hepatic function disorder was determined. Patients’ demographic and clinical characteristics are summarized using descriptive statistics, including frequencies and percentages for categorical data and median (range) for continuous data. A univariate Cox proportional hazards model (with time from the start of treatment to the onset of the AE) was used to identify background factors associated with AEs of hepatic function disorder, and calculate the hazard ratios with 95% confidence intervals. All statistical analyses were performed using SAS software version 9.4 (SAS Institute Inc., Cary, NC, USA).

## Results

### Patient characteristics

**Fig. 1:**
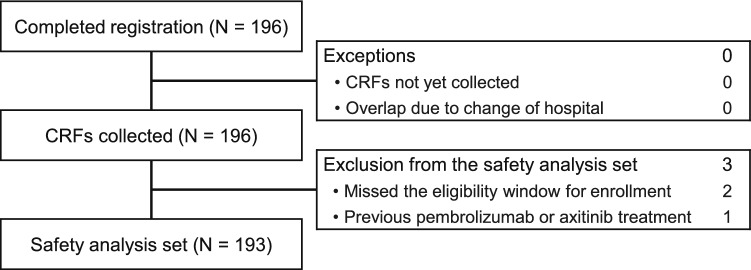
Patient disposition. CRF, case report form

Patient disposition is shown in Fig. 1. Among the 196 patients from whom survey forms were collected, 193 were included in the safety analysis set. Patient background characteristics are summarized in Table 1. Most patients (76.2%) were male. Furthermore, most patients (78.2%) had a Karnofsky Performance Status (KPS) of 80–100%, and 15.0% had a KPS ≤ 70%; the KPS was unknown in 6.7% of patients. The International Metastatic RCC Database Consortium risk classification [26] was favorable, intermediate, and poor in 20.2%, 58.6%, and 19.7% of patients, respectively. The percentage of patients who reported alcohol consumption was 33.7%, and that of patients with liver metastasis was 9.3%. Most patients (96.4%) did not have a history of hepatic function disorder before starting treatment.

**Table 1:** Patient background characteristics

Characteristics	N = 193
Age	
Median (range), years	70.0 (39–94)
≥ 65 years	130 (67.4)
≥ 75 years	49 (25.4)
Sex	
Male	147 (76.2)
Female	46 (23.8)
Karnofsky Performance Status	
80% to 100%	151 (78.2)
≤ 70%	29 (15.0)
Unknown	13 (6.7)
Time from diagnosis^a^	
< 1 year	128 (66.3)
1 year to < 3 years	19 (9.8)
≥ 3 years	39 (20.2)
Unknown	7 (3.6)
Stage	
Stage III	10 (5.2)
Stage IV	183 (94.8)
IMDC prognostic risk	
Favorable	39 (20.2)
Intermediate	113 (58.6)
Poor	38 (19.7)
Unknown	3 (1.6)
BMI	
< 25	140 (72.5)
≥ 25	47 (24.4)
Unknown/blank	6 (3.1)
Smoking history	
No	73 (37.8)
Yes, current	20 (10.4)
Yes, former	76 (39.4)
Unknown/blank	24 (12.4)
Brinkman index	
< 600	113 (58.6)
≥ 600	41 (21.2)
Unknown/blank	39 (20.2)
Alcohol consumption	
No	85 (44.0)
Yes	65 (33.7)
Unknown/blank	43 (22.3)
Liver metastases	18 (9.3)
Prior surgery^b^	102 (52.9)
Nephrectomy	95 (49.2)
Metastasectomy	14 (7.3)
Other	3 (1.6)
Prior radiation	17 (8.8)
Prior anticancer treatment	
No	187 (96.9)
Yes	4 (2.1)
TKI	1 (0.5)
IFN-α	2 (1.0)
Other	1 (0.5)
Unknown	2 (1.0)
ALT	
< 40 IU/L	182 (94.3)
≥ 40 IU/L	11 (5.7)
AST	
< 40 IU/L	181 (93.8)
≥ 40 IU/L	12 (6.2)
T-Bil	
< 1.5 mg/dL	188 (97.4)
≥ 1.5 mg/dL	4 (2.1)
Unknown	1 (0.5)
ALP	
< 350 IU/L	175 (90.7)
≥ 350 IU/L	17 (8.8)
Unknown	1 (0.5)
γ-GTP	
≤ 100 IU/L	165 (85.5)
> 100 IU/L	21 (10.9)
Unknown	7 (3.6)
Hepatic function disorder before treatment initiation	
No	186 (96.4)
Yes	6 (3.1)
Child–Pugh Grade A	5/6 (83.3)
Child–Pugh Grade B/C	0
Unknown	1/6 (16.7)
Unknown	1 (0.52)

Data are n (%) unless otherwise stated

ALP: alkaline phosphatase; ALT: alanine aminotransferase; AST: aspartate aminotransferase; BMI: body mass index; γ-GTP: gamma-glutamyl transpeptidase; IFN-α: interferon alpha; IMDC: International Metastatic Renal Cell Carcinoma Database Consortium; T-Bil: total bilirubin; TKI: tyrosine kinase inhibitor

^a^Time from diagnosis of renal cell carcinoma to initiation of treatment with pembrolizumab

^b^Some patients overlapped regarding the type of surgery received

### Treatment status

The treatment profile and treatment status of patients are summarized in Table 2. The median treatment period was 27.1 weeks, and the median number of pembrolizumab treatment cycles was six. At the data cut-off (9 months after starting treatment with pembrolizumab plus axitinib), the percentage of patients who continued treatment was 37.8% (73/193) and that of patients who discontinued treatment was 62.2% (120/193). The most common reason for treatment discontinuation was any AE in 31.1% (60/193) of patients, followed by disease progression in 15.0% (29/193) and death in 9.8% (19/193); some patients discontinued for multiple reasons.

**Table 2:** Treatment profile and treatment status

	N = 193
Treatment period, weeks	27.1 (0.1–36.0)
Observation period, weeks	36.0 (0.1–86.3)
Treatment profile^a^	
Pembrolizumab	
Number of treatment cycles	6 (1–12)
Axitinib	
Treatment duration, days	157.0 (3–257)
Initial dose per day, mg	10.0 (4–20)
Average dose per day, mg	8.9 (3.0–20.0)
Treatment status^a^	
Continued^b^	73 (37.8)
Discontinued	120 (62.2)
Any adverse event^c^	60 (31.1)
Disease progression^c^	29 (15.0)
Death^c^	19 (9.8)
Other^c^	12 (6.2)
Surgical intervention^c^	9 (4.7)
Lost to follow-up^c^	2 (1.0)

Data are n (%) or median (range)

^a^At the data cut-off (9 months after starting treatment with pembrolizumab plus axitinib)

^b^Continued use of either drug or both drugs

^c^Includes adverse events other than hepatic function disorder. Some patients overlap multiple reasons

### Outcomes

The incidences of AEs of hepatic function disorder by grade and causal relationship to treatment are summarized in Table 3. The incidence of AEs of hepatic function disorder of any grade was 30.1% (58/193 patients) and that of grade ≥ 3 AEs of hepatic function disorder was 15.0% (29/193 patients). The incidence of AEs of hepatic function disorder for which a causal relationship could not be ruled out by the study investigator was 81.0% for pembrolizumab (47/58 patients) and 84.5% for axitinib (49/58 patients).

**Table 3:** Incidences of AEs of hepatic function disorder by grade and causal relationship to treatment

AE of hepatic function disorder	N = 193
Any AEs	58 (30.1)
By grade	
Grade 1–2	28 (14.5)
Grade 3–4	29 (15.0)
Grade 5	0
Unknown	1 (0.5)
By causal relationship to the study drugs^a^	
Pembrolizumab	47/58 (81.0)
Grade 1–2	22/58 (37.9)
Grade 3–4	24/58 (41.4)
Unknown	1/58 (1.7)
Axitinib	49/58 (84.5)
Grade 1–2	24/58 (41.4)
Grade 3–4	24/58 (41.4)
Unknown	1/58 (1.7)
Pembrolizumab and axitinib	41 (21.2)
Not related to either drug	3 (1.6)

Data are n (%)

AE: adverse event

^a^The relevance of each drug was determined by the study investigator as “can be ruled out” or “cannot be ruled out”

**Fig. 2:**
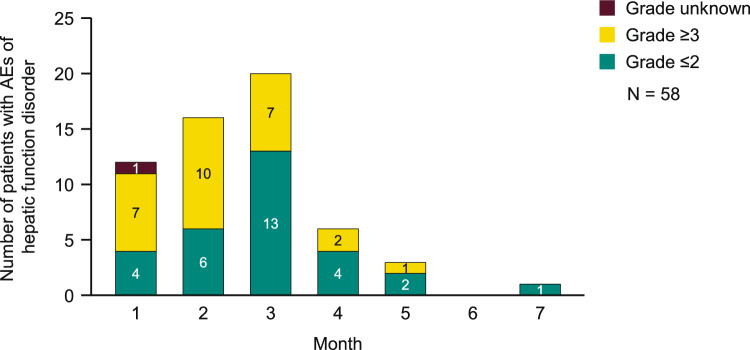
Number of patients with AEs of hepatic function disorder by month. AEs, adverse events

The numbers of patients with AEs of hepatic function disorder by month are shown in Fig. 2. Most AEs of hepatic function disorder occurred within 3 months (82.8% [48/58]) from initiation of treatment; however, an event occurred at 7 months after initiation of treatment.

**Fig. 3:**
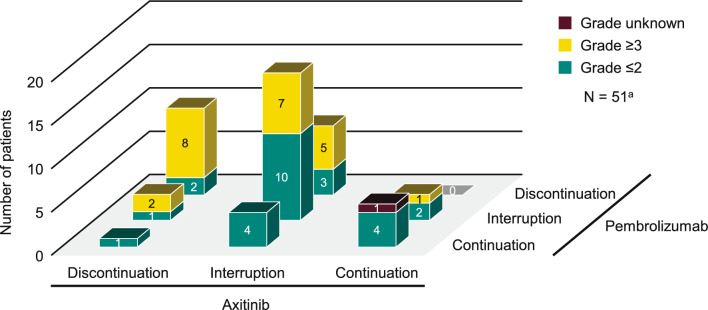
Dose continuations, interruptions, and discontinuations among patients with AEs of hepatic function disorder. ^a^Excluding 7 patients without treatment information from 58 patients with AEs of hepatic function disorder. AEs, adverse events

Continuations, interruptions, and discontinuations are shown in Fig. 3. AEs of hepatic function disorder resulted in discontinuation of pembrolizumab in 9.3% (18/193) of patients, axitinib in 7.3% (14/193) of patients, and both pembrolizumab and axitinib in 5.2% (10/193) of patients. Grade ≥ 3 AEs of hepatic function disorder resulted in discontinuation of pembrolizumab in 6.7% (13/193) of patients, axitinib in 5.2% (10/193) of patients, and both pembrolizumab and axitinib in 4.1% (8/193) of patients. The dose of axitinib was reduced (without discontinuation) because of an AE of hepatic function disorder in 10.4% (20/193) of patients.

Treatment interventions for AEs of hepatic function disorder are shown in Table 4. The percentage of patients who received corticosteroids for AEs related to hepatic function disorder was 6.7% (13/193); among the 13 patients who received corticosteroids, one patient received pulse corticosteroid therapy. After treatment with corticosteroids, the outcome was recovered or recovering in all patients. The proportion of patients who received ursodeoxycholic acid was 5.2% (10/193 patients), and that of glycyrrhizin was 3.1% (6/193 patients). No patients were treated with immunosuppressive drugs such as mycophenolate mofetil.

**Table 4:** Treatment interventions for AEs of hepatic function disorder AEs, adverse events

Safety analysis set, N = 193	Any grade n = 58	Grade 3–5^a^ n = 27
Corticosteroid	13 (6.7)	12 (6.2)^b^
Ursodeoxycholic acid	10 (5.2)	5 (2.6)
Glycyrrhizin	6 (3.1)	5 (2.6)
Mycophenolate mofetil	0	0
Azathioprine	0	0

Data are n (%). Percentages were calculated based on the total number of patients (N = 193). There were no grade 5 AEs

AEs: adverse events

^a^Maximum grade for each patient

^b^Including one patient who received steroid pulse therapy

**Fig. 4:**
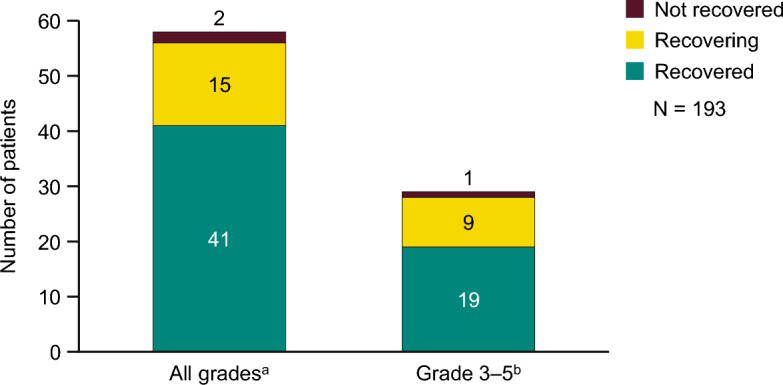
Outcomes of AEs of hepatic function disorder. ^a^In one patient who did not recover, the AEs of hepatic function disorder were of an unknown grade. ^b^There were no grade 5 AEs. There were no cases of “Death” or “Recovered with sequelae”. AEs, adverse events

Among the 58 patients with AEs of hepatic function disorder, the number of patients in whom the outcome was confirmed to be recovered or recovering was 56, and there were no deaths (Fig. 4). Cox proportional hazards model analysis did not identify any background factors with a lower limit of the 95% confidence interval > 1 and hazard ratio ≥ 2 (Online Resource 1).

## Discussion

In this PMS study, real-world data from 193 patients with unresectable/metastatic RCC in Japan confirmed the frequency of AEs of hepatic function disorder, and the details of 58 cases with AEs of hepatic function disorder were analyzed. The present study findings provide valuable information on the real-world safety of combination treatment with pembrolizumab and axitinib when used for the first time in patients with unresectable/metastatic RCC in Japan.

In the present study, patients were older and had more advanced malignancy (median age: 70 years, based on the International Metastatic RCC Database Consortium risk score: favorable risk in 20.2% of patients) than those enrolled in KEYNOTE-426 (62 years, 32%) [16]. Although the present study findings might reflect real clinical practice in Japan, the proportion of patients with extremely low KPS was small (KPS ≤ 70%: 15.0%).

There were no new safety signals for AEs of hepatic function disorder. The incidence of grade ≥ 3 AEs of hepatic function disorder (14.0% in the present study) was consistent with that previously reported in KEYNOTE-426 (21.2%; including grade ≥ 3 AEs of aspartate aminotransferase [AST] increased: 7.0%, alanine aminotransferase [ALT] increased: 13.3%, and hepatitis: 1.4%) [24], as well as that reported in the extended follow-up and a subset of Japanese patients (grade ≥ 3 TRAEs of ALT and AST increased in the overall population: 12.6% and 6.8%, respectively; grade ≥ 3 TRAEs of hepatic function abnormal in the subset of Japanese patients: 11.4%) [16, 17], a multicenter, retrospective study in real-world clinical practice in Japan (grade ≥ 3 TRAEs of abnormal hepatic function: 19.1%) [27], and in a phase 3 trial of non-Japanese patients (grade ≥ 3 TRAEs of ALT and AST increased: 12.1% and 6.8%, respectively) [15]. However, it tended to be higher than that reported previously for other ICI/TKI combination therapies, such as nivolumab plus cabozantinib (increased ALT 28.1% [any grade] and 5.3% [grade ≥ 3], increased AST 25.3% [any grade] and 3.4% [grade ≥ 3]) [10], avelumab plus axitinib (increased ALT 17.1% [any grade] and 6.0% [grade ≥ 3], increased AST 14.5% [any grade] and 3.9% [grade ≥ 3]) [11], and pembrolizumab plus lenvatinib (increased AST 11.1% [any grade] and 3.1% [grade ≥ 3], increased ALT 11.9% [any grade] and 4.3% [grade ≥ 3]) [12]. It should be noted that direct comparisons are difficult because the event categories corresponding to hepatic function disorder are different for each study, and it is not possible to directly compare the frequencies. No significant risk factors for AEs of hepatic function disorder were identified in the present PMS. The incidence of grade > 3 hepatic function disorder, an AEOSI, was 2.5% in a PMS of pembrolizumab monotherapy for urothelial carcinoma [22]. Overall, it is important to monitor patients treated with pembrolizumab as monotherapy or in combination with axitinib in accordance with the risk management plan [25].

Both ICI and TKI use have been associated with hepatotoxicity when administered as monotherapy in patients with cancer [28–31]. Axitinib-related toxicities occurring in patients with advanced RCC who are treated with an ICI drug plus axitinib can be effectively managed through axitinib modifications [32, 33]. It has been reported that the *UGT1A1* gene could be associated with hepatic function disorder in patients treated with the TKI pazopanib [34], but no such risk factors have been reported in patients treated with ICIs or axitinib. Although there were no grade 5 AEs in this PMS study, caution should be exercised because there have been reports of fatal cases of immune-related AEs in hepatic function due to the immunotherapy response in patients treated with ICI/ICI combination therapy [35].

The present study has some limitations. Considering that AEs other than hepatic function disorder were not included in this study, the occurrence of hepatic function disorder and the resumption/discontinuation of pembrolizumab plus axitinib cannot be directly linked. It was not possible to identify risk factors for the development of hepatic function disorder, possibly because of the small number of patients. In addition, the follow-up period was set at 9 months; we cannot rule out that some patients may have developed AEs outside this time period. Given that an AE of hepatic disorder occurred after 6 months in this study, careful longer-term observation is important.

In conclusion, there were no new safety signals for AEs of hepatic function disorder, and the incidence was consistent with that previously reported in KEYNOTE-426, in Japanese patients with radically unresectable/metastatic RCC treated with pembrolizumab plus axitinib in real-world clinical practice. Careful management and follow-up to evaluate the AEs of hepatic function disorder are needed for the appropriate use of combination treatment with pembrolizumab and axitinib.

## Supplementary Information

Below is the link to the electronic supplementary material.

Supplementary file 1 (PDF 218 KB) Online Resource 1. Background factors associated with AEs of hepatic function disorder for which a causal relationship could not be ruled out with pembrolizumab. For liver function tests (AST, ALT, ALP, γ-GTP, and T-Bil), blood samples were taken and analyzed before the first dose of treatment. AE, adverse event, ALP, alkaline phosphatase, ALT, alanine aminotransferase; AST, aspartate aminotransferase; BMI, body mass index; CI, confidence interval; γ-GTP, gamma-glutamyl transpeptidase; IMDC, International Metastatic RCC Database Consortium; KPS, Karnofsky performance status; T-Bil, total bilirubin.

## Data Availability

The data sets analyzed during this post-marketing surveillance are not available because data sharing with third parties is not permitted per the contract with all study sites or the patients. Please contact MSD K.K., Tokyo, Japan (https://www.msd.co.jp) for inquiries about access to the data set used in this post-marketing surveillance.
