# Supplementary material for: Adverse events of hepatic function disorder in Japanese patients with radically unresectable or metastatic renal cell carcinoma treated with pembrolizumab plus axitinib: a post-marketing surveillance study
Source: Int J Clin Oncol. 2025 Apr 29;30(6):1229–36. doi: 10.1007/s10147-025-02708-2 (PMC12122596; doi:10.1007/s10147-025-02708-2)

Electronic Supplementary Material

International Journal of Clinical Oncology

Adverse events of hepatic function disorder in Japanese patients with radically unresectable or metastatic renal cell carcinoma treated with pembrolizumab plus axitinib: a post-marketing surveillance study

Mototsugu Oya[1], Shotaro Yasuoka[2], Takuto Tokudome[2], Toshihiko Minegishi[2], Masahiro Hamada[3], Masahiko Ozaki[3], Shinichiroh Maekawa[3], Yuichiro Ito[2]

1. Department of Urology, Keio University School of Medicine, Tokyo, Japan
2. Oncology Medical Affairs, MSD K.K., Tokyo, Japan
3. Japan Pharmacovigilance, MSD K.K., Tokyo, Japan

Corresponding author

Takuto Tokudome

Address: Oncology Medical Affairs, MSD K.K., 1-13-12 Kudan-kita, Chiyoda-ku, Tokyo 102-8667, Japan

Tel: +81-3-6272-1000

E-mail: takuto.tokudome@merck.com

Online Resource 1. Background factors associated with AEs of hepatic function disorder for which a causal relationship could not be ruled out with pembrolizumab

For liver function tests (AST, ALT, ALP, γ-GTP, and T-Bil), blood samples were taken and analyzed before the first dose of treatment.

Abbreviations: AEs, adverse events; ALP, alkaline phosphatase; ALT, alanine aminotransferase; AST, aspartate aminotransferase; BMI, body mass index; CI, confidence interval; γ-GTP, gamma-glutamyl transpeptidase; IMDC, International Metastatic RCC Database Consortium; KPS, Karnofsky Performance Status; T-Bil, total bilirubin.

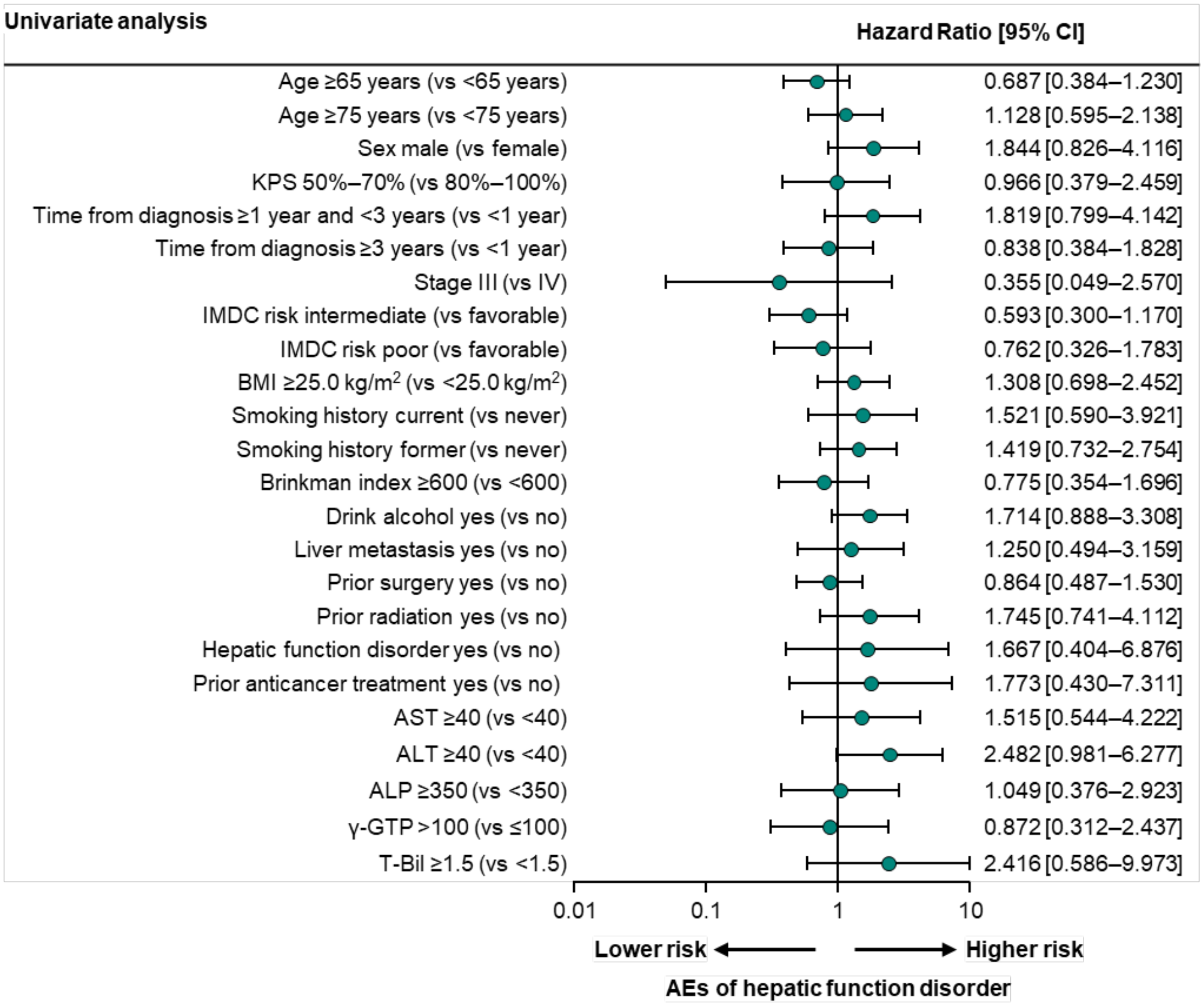

Supplement: Supplementary file 1 — (PDF 218 KB) Online Resource 1. Background factors associated with AEs of hepatic function disorder for which a causal relationship could not be ruled out with pembrolizumab. For liver function tests (AST, ALT, ALP, γ-GTP, and T-Bil), blood samples were taken and analyzed before the first dose of treatment. AE, adverse event, ALP, alkaline phosphatase, ALT, alanine aminotransferase; AST, aspartate aminotransferase; BMI, body mass index; CI, confidence interval; γ-GTP, gamma-glutamyl transpeptidase; IMDC, International Metastatic RCC Database Consortium; KPS, Karnofsky performance status; T-Bil, total bilirubin. [file 10147_2025_2708_MOESM1_ESM.pdf]
